# Usability and Acceptability of a Digital Health App to Support Infant Feeding and Lifestyle Behaviors: Mixed Methods Pilot Study

**DOI:** 10.2196/86139

**Published:** 2026-05-11

**Authors:** Madhumitha Ayyappan, Nowel Tan, Sarithra Jayasekaran, Chee Wai Ku, Chengsi Ong, Chua Mei Chien, Jerry Kok Yen Chan, Fabian Yap, See Ling Loy, Daniel Chan

**Affiliations:** 1 Duke-NUS Medical School, 8 College Rd, Singapore, 169857, Singapore, 65 93871908; 2 Department of Paediatrics, KK Women's and Children's Hospital, Singapore, Singapore; 3 Endocrinology Service, Department of Paediatrics, KK Women’s and Children’s Hospital, Singapore, Singapore; 4 Department of Reproductive Medicine, KK Women’s and Children’s Hospital, Singapore, Singapore; 5 SingHealth Duke-NUS Maternal and Child Health Research Institute, KK Women’s and Children’s Hospital, Singapore, Singapore; 6 Department of Nutrition and Dietetics, KK Women’s and Children’s Hospital, Singapore, Singapore; 7 Department of Neonatology, KK Women's and Children's Hospital, Singapore, Singapore; 8 Lee Kong Chian School of Medicine, Nanyang Technological University, Singapore, Singapore

**Keywords:** mobile health, mHealth, childhood obesity, obesity prevention, infant feeding, early-life behaviors, usability, acceptability, mobile apps

## Abstract

**Background:**

Childhood obesity is a global health concern with long-term cardiometabolic and psychosocial consequences. Establishing healthy feeding and lifestyle behaviors from infancy is critical to population health efforts with a life course perspective. Recently, digital health applications have gained traction in reaching out to parents and promoting healthy feeding behaviors.

**Objective:**

The aim of this study was to evaluate the usability and acceptability of the Feeding, Lifestyle, Activity Goals app in providing parents with evidence-based guidance and tailored advice to promote healthier eating behaviors and lifestyle habits among infants.

**Methods:**

We conducted a mixed methods pilot study among parents of infants aged 0 to 24 months recruited from a tertiary maternity and children’s hospital in Singapore. Participants used the digital health app for 7 days. Usability was assessed using the validated System Usability Scale (score range 0‐100, with ≥70 indicating acceptable usability). Acceptability was explored via open-ended questionnaires on satisfaction, relevance, and user experience, with qualitative data analyzed thematically by 2 independent coders to ensure rigor.

**Results:**

A total of 26 parents of infants aged 3 weeks to 23 months completed the study. The app demonstrated good usability, with a mean System Usability Scale score of 71.3 (SD 11.0). Parents valued its role in organizing infant care; promoting self-reflection on parenting practices; and providing personalized, evidence-based recommendations. These benefits were particularly appreciated by first-time parents and those with multiple caregiving responsibilities. Challenges included an unintuitive user interface, high manual data entry burden, and advisory content that was occasionally overly general or text heavy. These insights highlight clear priorities for onward efforts in optimization.

**Conclusions:**

Results of our mixed methods evaluation indicate that our digital health app demonstrated good usability and acceptability among parents of infants. Targeted refinements to the interface, data entry processes, and content delivery are warranted before large-scale evaluation to determine its impact on early-life health behaviors and obesity prevention.

## Introduction

Childhood obesity is rising at an alarming rate worldwide. According to the World Health Organization [1], an estimated 35 million children under the age of 5 years are overweight, and the prevalence of children and adolescents with overweight aged 5 to 19 years has risen significantly from 8% in 1990 to 20% in 2022. This surge in childhood obesity not only results in health-related comorbidities such as metabolic syndrome and orthopedic issues but also significantly affects children’s psychological well-being, body image, and overall quality of life [2]. In the long term, these children also have a moderate risk of obesity persisting into adulthood [3], along with its cardiovascular complications.

Numerous studies have shown that children’s eating habits [4] and food preferences [5] are strongly linked to their body composition, with unhealthy diets and poor feeding practices increasing the risk of childhood obesity. Importantly, these dietary patterns often persist into adulthood as feeding practices established in early life influence future dietary and taste preferences [6,7]. Therefore, timely interventions during early childhood, particularly those aimed at shaping healthy feeding behaviors, are critical for long-term obesity prevention and for promoting sustainable, healthy growth trajectories [8]. Parents play a central role in this process [9]. From infancy, they influence not only the types and amounts of food offered to children but also the emotional and environmental context in which meals take place [10]. As a result, many public health initiatives encourage parents to act as “health promoters” shaping their child’s feeding behaviors and long-term food preferences through informed and consistent modeling and support [11,12].

However, providing parents with consistent, evidence-based support remains challenging. Traditional interventions targeting feeding behaviors, such as in-person counseling and motivational interviewing, have shown benefit but are often labor-intensive, time-consuming, and difficult to scale [13]. With the advent of technological advancements, digital interventions such as mobile health (mHealth) apps have gained traction as a means of expanding outreach to parents and providing accessible, around-the-clock support for improving dietary choices and feeding behaviors [11]. For instance, a recent pilot study evaluating a parent-focused mHealth app designed to promote healthy eating behaviors in children found that parents perceived the app as helpful in increasing their nutritional knowledge and awareness of their children’s diet [12]. Similar digital interventions in adult populations have also shown promise, with meta-analyses highlighting their effectiveness in managing and preventing obesity [14]. However, despite these benefits, commercially available mHealth apps still lack medical oversight, are not co-designed by stakeholders including health care professionals and end users, and often fall short in providing evidence-based or culturally tailored content [15,16]. These drawbacks limit their effectiveness and credibility, particularly when used as stand-alone tools for behavior change. In addition, given that cultural influences strongly shape feeding practices [17], there is a clear need for digital solutions that are evidence based, medically guided, and culturally sensitive, with the eventual aim of reinforcing good dietary practices and habits in the pediatric population.

To this end, our study team involving a diverse panel of domain experts, including neonatologists, pediatricians, dietitians, speech therapists, physiotherapists, and nurses, developed a digital health app named Feeding, Lifestyle, Activity Goals (FLAGs) to promote healthier eating behaviors and lifestyle habits in infants from birth to the age of 2 years. Its development involved a meticulous process for which the questionnaires and advisory content underwent extensive curation [18]. The tool is grounded in theoretical frameworks such as the capability, opportunity, and motivation–behavior framework, which emphasizes enhancing parents’ capabilities through skill-building, while providing opportunities and motivation via a user-friendly app to support behavioral change and promote healthy feeding habits. Overall, FLAGs serves as both an assessment tool and a provider of tailored advice for parents on their infants’ feeding practices, sedentary behavior, and physical activity. In this study, we specifically aimed to perform a mixed methods evaluation of the app’s usability and acceptability among end users. These findings will inform iterative refinements and subsequent large-scale trials to examine the app’s efficacy in effecting behavior change and on anthropometric outcomes.

## Methods

### Study Design and Setting

We conducted a mixed methods pilot study at KK Women’s and Children’s Hospital, Singapore, to evaluate the app’s usability and acceptability among parents of infants. The study period was from September 2024 to November 2024.

### Participants and Recruitment

A purposive sampling strategy was used to recruit eligible parents or primary caregivers of infants aged 0 to 24 months during outpatient clinic visits. Sampling sought to capture diversity in parental age, ethnicity, and caregiving experience to obtain a broad range of perspectives on usability and acceptability.

Our inclusion criteria were infants aged 0 to 24 months as well as parents’ ability to understand a basic level of English in view of the fact that all the content within the app and the feedback instruments used was in English. Meanwhile, our exclusion criteria were any infants with congenital abnormalities and physical or neurodevelopmental disabilities that may interfere with app use or who may have unique feeding requirements.

### Ethical Considerations

Ethics approval was obtained from the SingHealth Centralised Institutional Review Board, Singapore (reference 2024/3224). The study was conducted in accordance with the principles of the Declaration of Helsinki and adhered to all applicable local regulatory requirements. All participants received a verbal and written explanation of the study and provided written informed consent before enrollment. Upon successful completion of the study, participants received reimbursement of SG $20 (approximately US $15). Each participant was assigned an anonymized study ID; all collected data were encrypted and stored on a secure, password-protected server accessible only to the study team.

### Study Procedures

At baseline, we collected parental and infant demographic data, including age, sex, ethnicity, and gestational age at birth. Infant anthropometry was measured using the Seca 210 mobile measuring mat for recumbent length and Seca 334 weighing scale for weight, and BMI-for-age was calculated as weight divided by height squared (kg/m^2^). After recruitment, participants underwent a cognitive walk-through via both verbal explanation and a pictorial guide demonstrating the user interface with key features. They were then provided with installation instructions and granted full access to the app for a 7-day trial. In terms of the duration for this trial, a study by Steele et al [19] in Durham, North Carolina, United States, found that brief intensive internet-delivered cognitive behavioral therapy on social anxiety for 7 days was feasible, with low premature dropout rates. Similarly, a 7-day mobile tracking study [20] on sleep in students found that there was sufficient variance in terms of the data provided. Hence, the decision was made to use a 7-day trial.

Participants were encouraged to explore all functions without a prescribed recording schedule to reflect real-world use. Intake data (feeding, activity, and growth) could be logged ad libitum. Following the 7-day trial, participants promptly provided an interview time. During the online video interview, the System Usability Scale (SUS) questionnaire was administered via screen sharing, and responses were recorded accordingly. Subsequently, participants continued in a semistructured interview format with open-ended questions. Interviews were audio recorded with consent and transcribed verbatim. All interviews were completed within 1 month from the time of recruitment.

### FLAGs Digital Health App

To address the need for an optimized, medically guided feeding intervention, our team at KK Women’s and Children’s Hospital first developed a validated assessment instrument—the FLAGs digital health app—to evaluate infant feeding and lifestyle behaviors. The team comprised neonatologists, pediatricians, obstetricians, and dietitians. Psychometric evaluation of the app revealed strong clarity, relevance, and simplicity. Overall, the Cronbach α was 0.71, and the intraclass correlation coefficient of 0.861 (*P*<.001) indicated statistically significant test-retest reliability, supporting the instrument’s validity and reliability [18]. Subsequently, the instrument was converted into a digital health app that enables caregivers to log and track their infants’ dietary intake, physical activity, and growth parameters, generating visual summaries (graphs and trends) and personalized, evidence-based recommendations. Another framework that provided a foundation for our digital health app was the theory of planned behavior [21]. This theory looks at the close relationship between infant feeding practices and parents’ behaviors, which are shaped by subjective norms and perceived control.

**Figure 1.**
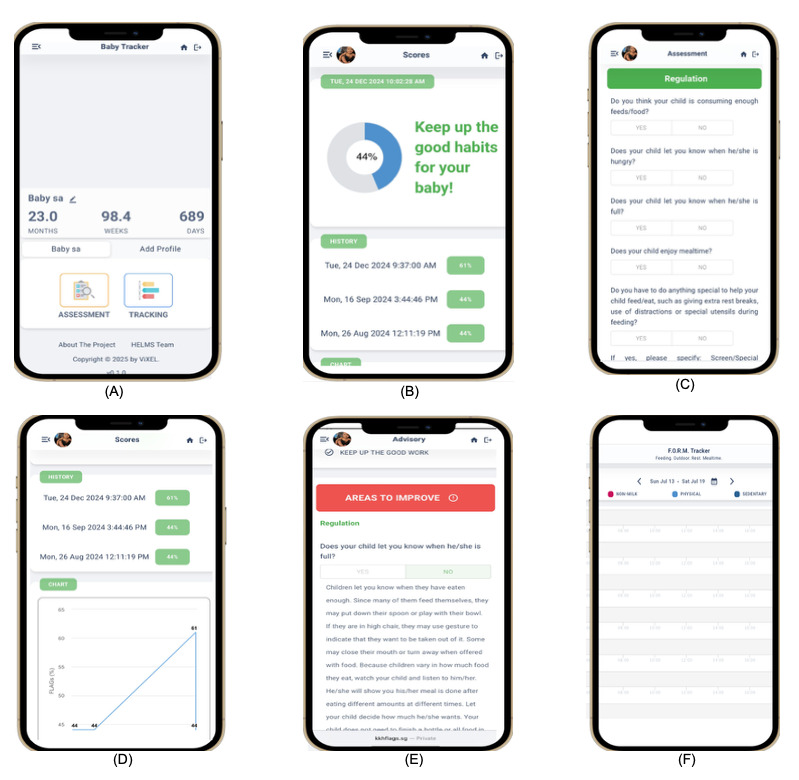
Screenshots of the Feeding, Lifestyle, Activity Goals (FLAGs) digital health app: (A) main landing page, (B) score display screen, (C) FLAGs questionnaire, (D) trends in feeding and lifestyle scores, (E) tailored parental advisory and recommendations, and (F) daily feed and physical activity log.

The app consists of several user modules, beginning with a secure log-in and profile setup that personalizes questions based on the child’s age and feeding stage. The assessment module comprises 11 to 18 items categorized into 3 core domains: regulation, which assesses recognition of hunger and satiety cues, feeding responsiveness, and infant self-soothing; weaning, which covers complementary feeding initiation, food texture progression, and parental feeding approaches; and habituation, which evaluates sleep duration, screen exposure, and daily activity routines. Each question is age specific and uses either a binary (“yes” or “no”) or categorical (eg, 5 to 30 minutes per meal or ≤1 hour vs >1 hour of screen time) response format. Responses are automatically scored within the app: a healthy, age-appropriate behavior is awarded 1 point, whereas an at-risk or suboptimal response receives 0 points. Domain-specific subscores and an overall FLAGs composite score (0%‐100%) are then computed in real time. Upon scoring, the app instantly generates personalized advisory feedback displayed on the “Advisory” and “Scores” pages, providing parents with clear, actionable recommendations to support healthy feeding and lifestyle practices. The app also features daily and weekly tracking modules that allow users to record milk intake, food consumption, physical activity, sedentary time, and sleep. Input forms are editable for flexibility, with weekly summaries enabling trend visualization over time. The behavioral nudges within the FLAGs app are automatically tailored to the child’s age group and corresponding developmental stage. Key interface elements of the intervention are shown in Figure 1.

### Outcomes and Measures

The primary outcome was usability, assessed using the validated 10-item SUS [22] (score range 0‐100, with ≥70 indicating acceptable usability for digital tools). This was interviewer administered, with a standardized script for each question; no prompts, explanations, or leading cues were provided beyond the original wording of the SUS items, and participants were given time to consider each question and respond accordingly.

The secondary outcome was acceptability, assessed via open-ended, semistructured interviews. Participants were asked the following five questions: (1) “what did you like about the digital health application?” (2) “What did you dislike about the digital health application?” (3) “What features would you like to see retained in the digital health application?” (4) “What features would you like to remove or change in the digital health application?” (5) “Do you have any other comments about the digital health application?” These questions were designed to elicit both positive and negative experiences, feature-specific feedback, and additional suggestions for improvement. An interview guide that includes the SUS and acceptability questionnaire used is provided in Multimedia Appendix 1.

### Data Analysis

Quantitative data analysis was conducted using Stata (version 18; StataCorp LLC). Participant demographic and baseline characteristics were tabulated to provide a descriptive overview of the sample. For the SUS, the mean and SD, as well as sample size and frequency, were calculated for each individual item to identify variability in responses and for the total SUS score to assess overall system usability.

To qualitatively analyze user experience of the FLAGs app, all audio recordings of participant interviews were transcribed and analyzed using Microsoft Word. Using the grounded theory approach [23], data analysis involved line-by-line coding, axial coding, and selective coding by 2 independent researchers to generate broad conceptual themes. In the later stage of analysis, all major categories, themes, and subthemes were operationalized. Relationships between themes and subthemes were proposed and substantiated with relevant quotes from participants. To ensure research rigor and trustworthiness of the findings, stringent mechanisms were put in place. These included maintaining an audit trail of research decisions, changes, and data analysis; interresearcher consensus in finalizing themes; and frequent peer debriefing to review interpretations and findings so as to minimize researcher bias [24,25].

## Results

### Participant Characteristics

**Figure 2.**
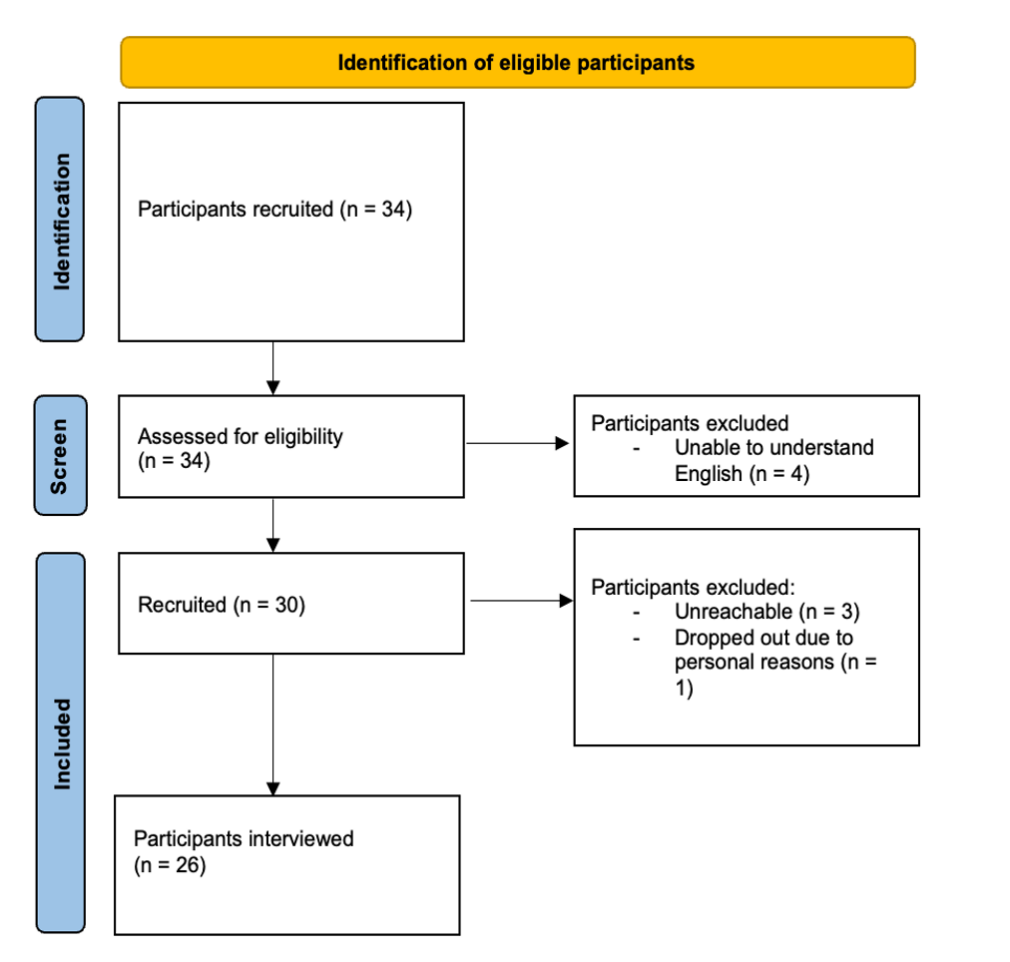
Flowchart of participant selection for the Feeding, Lifestyle, Activity Goals digital health app study.

A total of 34 participants were recruited and assessed for eligibility for the study. Subsequently, of the 34 assessed participants, 11.8% (4/34) were excluded as they were unable to understand English, which is the working language of the app and survey questionnaires. Eventually, of the remaining 30 participants, 86.7% (26/30) were interviewed as 10% (3/30) were lost to follow-up and 3.3% (1/30) withdrew from the study for personal reasons. Figure 2 shows the flowchart of the included and excluded participants and those who completed the study.

Table 1 describes the baseline characteristics of the parents and their children. Of the 26 parents, 73.1% (19/26) were female, and the mean age was 33.7 (SD 5.2) years. In terms of educational level, most parents were diploma holders (10/26, 38.5%) and degree holders (10/26, 38.5%). Regarding the demographic characteristics of the children, 53.8% (14/26) were males. The mean age was 7.4 (SD 5.0) months, with ages ranging from 3 weeks to 23 months.

**Table 1.** Baseline characteristics of the participants (N=26).

Characteristics	Participants
Caregiver’s age (y), mean (SD)	33.7 (5.2)
Caregiver’s relationship to the child, n (%)	
Mother	19 (73.1)
Father	7 (26.9)
Caregiver’s monthly household income, n (%)	
<SGD 10,000 (US $7830.60)	17 (65.4)
≥SGD 10,000 (US $7830.60)	9 (34.6)
Child’s age (mo), mean (SD)	7.4 (5.0)
<12, n (%)	18 (69.2)
≥12, n (%)	8 (30.8)
Child’s sex, n (%)	
Male	14 (53.8)
Female	12 (46.2)
Child’s ethnicity, n (%)	
Chinese	13 (50)
Indian	9 (34.6)
Malay	4 (15.4)
Child’s gestational age at birth (wk), n (%)	
Full term (≥37)	21 (80.8)
Preterm (<37)	5 (19.2)

### Usability

Table 2 shows the proportion of responses and the mean scores for questions from the SUS. Scores for each item on the questionnaire range from 1 to 10, with a maximum possible score of 100. The mean SUS score for our FLAGs app was 71.3 (SD 11.0). Half (14/26, 53.8%) of the participants reported that they would use this digital intervention frequently to improve their children’s feeding behavior and nutritional choices. In addition, more than two-thirds (19/26, 73.1%) thought that the product was easy to use. Most (21/26, 80.8%) disagreed that they required technical assistance to use the digital intervention. Overall, this reported ease of use of the product appeared to translate into confidence in the implementation of the app into their lifestyles (20/26, 76.9%).

**Table 2.** Breakdown of responses to the System Usability Scale (SUS) questionnaire. The SUS score was calculated based on processes originally developed by Brooke [26] (N=26).

	Score, mean (SD)	Disagree, n (%)	Neutral, n (%)	Agree, n (%)
I think that I would like to use this product frequently.	3.4 (1.2)	6 (23.1)	6 (23.1)	14 (53.8)
I found the product unnecessarily complex.	2.6 (1.1)	17 (65.4)	2 (7.7)	7 (26.9)
I thought the product was easy to use.	4 (1.0)	3 (11.5)	4 (15.4)	19 (73.1)
I think that I would need the support of a technical person to be able to use this product.	1.7 (1.0)	21 (80.8)	3 (11.5)	2 (7.7)
I found the various functions in the product were well integrated.	3.6 (0.9)	4 (15.4)	7 (26.9)	15 (57.7)
I thought there was too much inconsistency in this product.	2.0 (0.7)	7 (26.9)	12 (46.2)	7 (26.9)
I imagine that most people would learn to use this product very quickly.	4.2 (0.9)	1 (3.8)	3 (11.5)	22 (84.6)
I found the product very awkward to use.	2.3 (1.0)	17 (65.4)	6 (23.1)	3 (11.5)
I felt very confident using the product.	4.0 (0.8)	1 (3.8)	5 (19.2)	20 (76.9)
I needed to learn a lot of things before I could get going with this product.	2.2 (1.1)	15 (57.7)	8 (30.8)	3 (11.5)

### Acceptability

#### Overview

The acceptability of the FLAGs app was evaluated through qualitative responses from parents regarding their experiences using the digital tool. These responses were categorized into three overarching themes: (1) positive experiences, (2) challenges encountered, and (3) desired features not currently available. Each theme was further broken down into subthemes reflecting specific aspects of the user experience.

#### Theme 1: Positive Experiences

This theme highlights the perceived benefits of using the FLAGs app, particularly in enhancing the organization of infant care, improving self-awareness and reflection, and fostering parental empowerment. These strengths suggest strong acceptability of the app among parents and caregivers.

##### Better Organization of Infants’ Care

Parents reported that the app supported better management of their infants’ daily routines, particularly regarding feeding and sleeping schedules. This benefit was especially prominent among first-time parents or those with multiple responsibilities:

The tracker is very useful, and it helps me to see if my baby had fed or not. I don’t have to keep recalling when I feed my baby.

[P7]

I’m more aware of the baby and I am very interested to log the feeding habits of baby into the app.

[P6]

##### Greater Clarity on Parenting Practices

The app’s personalized assessments and data visualization features allowed users to reflect on their parenting practices and identify areas for improvement. This was facilitated through automated, tailored feedback based on questionnaire responses:

Overall, the app is good to use but there are still more apps like this, but this is special because [it has] the advisory based on what you answer in the questionnaire.

[P1]

The assessment also allowed parents to reflect and self-evaluate while visualizing their progress over time through graphics such as bar graphs and charts:

The advisory is very helpful so it can show me roughly where I stand in terms of what to improve as a parent for my baby.

[P7]

##### Empowerment in Infant Care

Parents expressed that the app supported evidence-based parenting while reducing their dependence on health care providers for day-to-day concerns:

I like the fact that this app is like a guide. Like for example, they will tell you what is good and what is not good. For example, I can know how long of a nap is good for my baby.

[P5]

As (a) first-time parent I feel like I don’t need to seek for doctors help or ask doctors on a regular basis.

[P11]

##### Other Strengths

Other less common but pertinent strengths included the fact that the app was supported by KK Women’s and Children’s Hospital, a public tertiary maternal-child hospital, thus improving parental trust:

Maybe there are more apps, but this is maybe more trustworthy because it’s from [a] hospital.

[P8]

Others appreciated the delivery of behavioral nudges via WhatsApp:

I quite like the concept of this whole thing [the app] including the nudges which is a fresh idea to be sent [through] WhatsApp.

[P9]

### Theme 2: Challenges Encountered

Despite the positive experiences, several challenges with the app were identified, primarily related to usability—including operational issues, the complexity of the tracking process, and the relevance of the content—which helped further contextualize the SUS score.

#### Further Improvements Required in the User Interface

Many users described the app as being visually unrefined and having navigation issues. Issues ranged from log-in troubles to technical bugs:

For the login page, I had to keep logging in for the 7 days and I find it a bit troublesome and time consuming and there is no password view option, so I took very long to login to the app initially.

[P9]

When I did the assessment, I cannot look back at it after I complete it. Like there is no record of my assessment. When I did it one time, I cannot see the output of like the results of it. I briefly saw the results but when I tried to find it again, it is disappeared.

[P8]

#### Cumbersome Tracking Process

Users expressed difficulty in consistently inputting feeding and sleep data, especially when multitasking or without prompts from the app:

Since I do not really know the exact timing of the baby sleeping and feeding time I can’t really be consistent with the app. I just try my best when I’m at home to try login. Also, when I’m helping to take care of the baby I try to put away my phone from sight, so the baby doesn’t try to take it. So, I can use only based on my memory.

[P18]

This was especially relevant as the app did not auto-suggest entries based on previous entries, thereby increasing the cognitive load on parents:

I feel like the macro of the food is necessary information for medical side but for me I feel stressed to put in so many things. [The app they were using previously] is better comparatively because I can choose the food from previous options I have keyed in the app.

[P11]

#### Wordy Content

The app’s advisory section was seen as too text heavy, with users preferring more concise and targeted information:

I think I find it very wordy generally. There are more things to input, and the advisory is also very wordy. I think bite sized information is preferred by most of the parents because everything is fast moving now. And maybe if user can choose like a certain topic that they are more interested then, it can give a full explanation. Like if I’m worried about my baby’s sleep then I can click on the sleep advisory for more details.

[P9]

Furthermore, some feedback suggested that the assessments could be misleading or stressful due to a lack of sufficient personalization:

“Your child doesn’t need any distraction or effort when feeding.” That was not true for our child who really did not enjoy mealtimes despite countless efforts. [The recommendations] should be stated clearly that it may not always be the case.

[P27]

### Theme 3: Desired Features Not Currently Available

Several participants proposed enhancements to improve the app’s usability and collaborative potential. These fell into 3 subcategories: shared access, simplified input methods, and better access to historical data.

#### Shared Access for Different Caregivers

Users expressed a desire for options for multiple caregivers, emphasizing the importance of collaborative tracking. Suggestions included a shared account for caregivers and ease of log-in for multiple users:

And it will provide better communication between caretakers.

[P12]

I try to login using my husband phone also so I use only evening time. So I could not every time login the food and sleep. I just put in what I remember.

[P17]

#### Simplified Input Methods

Participants expressed a need for more intuitive input methods, such as preset options, visual timers, and icons to reduce manual entry burden:

As for the inputting the details of the data, it could be easier. To be honest, because I am using another app, I haven’t been using it regularly also but whenever I get the time I use it. So even for that app when it’s easier to type the data, I don’t seem to use it often, so this app I don’t see myself being very frequently using it. In terms of icon, there can be a new icon for breast feeding to show like breast milk vs formula. It’s like for easier navigation so I don’t have to type out what type of milk I’m feeding all the time. Inputting the timing is also quite tedious, instead they can have like a stopwatch like the baby time app I’m using now. There is an instant update of the current time the mummy Is feeding.

[P21]

Photo uploads were another frequently requested feature, especially for food tracking and medical documentation:

It’s easier to upload a picture and will be easier for your reference as well. And when I want to show let’s say to my doctor also, he can get the idea of how the food actually looks.

[P12]

Good to also include option to upload photos...i.e. stool consistency and color.

[P22]

#### Archiving and Access to Past Assessments

A common request was for a feature that allows users to revisit previous assessments and advisories. This allows for longitudinal tracking and review of prior advisories and scores:

I want to look back at the advisories and maybe have like an archive of previous advisories and assessment scores so that we can see the improvement and refer easily to the advice.

[P6]

## Discussion

This study evaluated the usability and acceptability of an mHealth app designed to support parents of infants aged 3 weeks to 23 months with evidence-based resources and personalized advice on feeding, physical activity, and growth. Overall, the digital health app demonstrated good usability, as evidenced by the mean SUS score. This places FLAGs in a range consistent with functional but improvable health technology [19]. In terms of acceptability, parents emphasized several positive aspects of the app, suggesting that it was generally well accepted within the study population. In particular, parents valued the structured organization of caregiving tasks, the ability to visualize progress, and the provision of evidence-based feedback in real time. These features created a sense of confidence and empowerment, particularly for first-time parents and for those balancing multiple caregiving roles. Many participants expressed that FLAGs offered reassurance and guidance that might otherwise be unavailable between clinical encounters, reflecting both the app’s acceptability and its potential to bridge gaps in routine parenting support. Challenges, particularly on the usability front, still remain and will guide subsequent work to improve the app’s usability. For example, the interface was perceived to be occasionally unintuitive, and data entry, while comprehensive, was regarded as burdensome and overly manual, limiting sustained engagement in day-to-day use. Advisory content was sometimes experienced as verbose, which risked diluting its impact and reducing its immediate applicability. Frequently suggested improvements included the ability to share access across multiple caregivers, streamlined logging features such as photo uploads and visual timers, and the capacity to archive and retrieve past assessments. Taken together, these findings suggest that FLAGs has achieved sufficient baseline usability and acceptability to justify further development, with a road map for further iteration.

When considered in relation to the wider literature, our findings reinforce both the promise and the challenges of parent-facing digital health interventions in early childhood, consistent with evidence from preschool interventions. For example, the MINISTOP (Mobile-Based Intervention Intended to Stop Obesity in Preschoolers) trial demonstrated improvements in composite diet and activity behaviors among children aged 4 years, underscoring the feasibility of app-based parent engagement [13]. Similarly, the Food4toddlers study revealed that parental access to the Food4toddlers website improved the frequency of intake of vegetables among toddlers immediately after the 6-month intervention period ended [27]. Building on these works, the Early Food for Future Health trial noted that an internet-based tool for parents of children aged between 6 and 12 months was appropriate and feasible to propagate information on healthy infant feeding to Norwegian mothers, leading to an increase in young children’s daily vegetable and fruit intake [28]. More generally, meta-analyses confirm that digital health interventions can promote healthier nutrition behaviors in children and families [11] and are effective in weight management among adults [14]. At the same time, reviews of pediatric nutrition apps have raised concerns regarding lack of clinical input, inadequate behavior change mechanisms, and poor usability [15,16]. A scoping review on child health promotion apps by Blakeslee et al [29] similarly acknowledged the importance for apps to be grounded in transparent theoretical frameworks and that content should be tailored to include intuitive and adaptive features—all challenges mirrored by parents’ feedback in our study calling for clearer, more targeted content and more usable logging. These findings highlight a key point: effectiveness is unlikely to be determined by the presence of digital content alone but rather by the integration of evidence-based content, clinical oversight, and user-centered design.

Our findings carry several implications for future iterations of FLAGs and design of similar interventions. First, minimizing interaction burden is likely to be critical for engagement. Parents consistently requested features that reduce repetitive manual data entry, such as preset options for routine inputs, auto-suggestions, and smart defaults [20,21]. Incorporating semipassive data capture where feasible could further enhance efficiency and lower friction. Second, personalization emerged as a strong driver of acceptability. Tailoring advisory content to infant age, feeding stage, and parent preferences and delivering messages in concise, layered formats would likely improve both perceived relevance and usability [29,30]. Third, supporting the broader caregiving ecosystem will be essential. Parents emphasized the need for shared caregiver accounts, secure photo uploads, and archival functions that support continuity of care, reflecting the reality that infant caregiving is rarely the responsibility of a single individual. Embedding FLAGs into routine pediatric follow-up visits such as growth monitoring or immunization appointments may normalize its use and provide opportunities for light-touch reinforcement by clinicians without imposing additional workload. Finally, equity considerations must be foregrounded in subsequent iterations, including the development of multilingual content, culturally appropriate guidance, offline-tolerant features, and accessible visual design.

Moving forward, our study team intends to evaluate the efficacy of the FLAGs app through a randomized controlled trial (NCT06457750) in improving anthropometric outcomes and establish its broader behavioral and health impacts [30]. This prospective interventional study aims to examine changes in infant feeding practices; mealtime routines; and anthropometric outcomes such as weight-for-length, BMI, and adiposity. Incorporating validated behavioral scales such as the Baby Eating Behavior Questionnaire and Children’s Eating Behavior Questionnaire [31] alongside growth metrics would provide a robust assessment of efficacy. Equally important will be the systematic evaluation of mediators (such as engagement dose and message exposure) and moderators (such as parity and baseline feeding difficulties). Use analytics, including log-in frequency, feature taps, and completion of advisories, should be analyzed a priori to explore “dose-response relationships” and fidelity of engagement [20,21,30]. Such an approach would advance understanding of not only whether the app works but also how it works and for whom it is most effective.

This study has several strengths. The FLAGs digital health app was developed through a clinically informed, framework-based process integrating evidence-based recommendations with input from health care professionals. The mixed methods design is a comprehensive process of evaluation that allowed for triangulation of quantitative and qualitative insights, enriching interpretation and ensuring that usability ratings were contextualized by lived experiences. Rigorous qualitative analysis procedures, including independent coding, consensus discussions, and audit trails, enhanced the trustworthiness of the findings.

Nevertheless, several limitations should be acknowledged. The sample was small, clinic based, and restricted to English-speaking participants, which limits generalizability. The 7-day evaluation period was limited in duration and did not permit the assessment of long-term engagement or sustained behavioral effects. Outcomes relied primarily on self-report, introducing potential recall and social desirability biases, whereas the clinic-based recruitment may have contributed to selection bias. These constraints are typical of a pilot study but must be addressed in future work. Larger and more diverse samples; extended follow-up; and incorporation of objective measures, including food photo diaries and automated tracking of physical activity, will be useful in validating these findings and evaluating real-world impact.

In conclusion, our pilot study showed that FLAGs, a medically guided digital health app, was deemed to be usable and acceptable by target end users, providing structured, evidence-based support for infant feeding and lifestyle practices while identifying areas for refinement such as data entry, personalization, and multicaregiver access. With further system and interface optimization, as well as robust evaluation of its efficacy in a subsequent interventional study, our tool has potential as a scalable strategy to promote healthy routines in early life and support obesity prevention.

## Supplementary material

10.2196/86139 Multimedia Appendix 1 Interview guide including the System Usability Scale and acceptability questionnaire.

## Abbreviations

FLAGs: Feeding, Lifestyle, Activity Goals
mHealth: mobile health
MINISTOP: Mobile-Based Intervention Intended to Stop Obesity in Preschoolers
SUS: System Usability Scale
