# Supplementary material for: Usability and Acceptability of a Digital Health App to Support Infant Feeding and Lifestyle Behaviors: Mixed Methods Pilot Study
Source: JMIR Pediatr Parent. 2026 May 11;9:e86139. doi: 10.2196/86139 (PMC13160257; doi:10.2196/86139)
Supplement: Multimedia Appendix 1 [file pediatrics-v9-e86139-s001.docx]

**Multimedia Appendix 1 – interview guide on the System Usability Scale (SUS) and acceptability questionnaires**


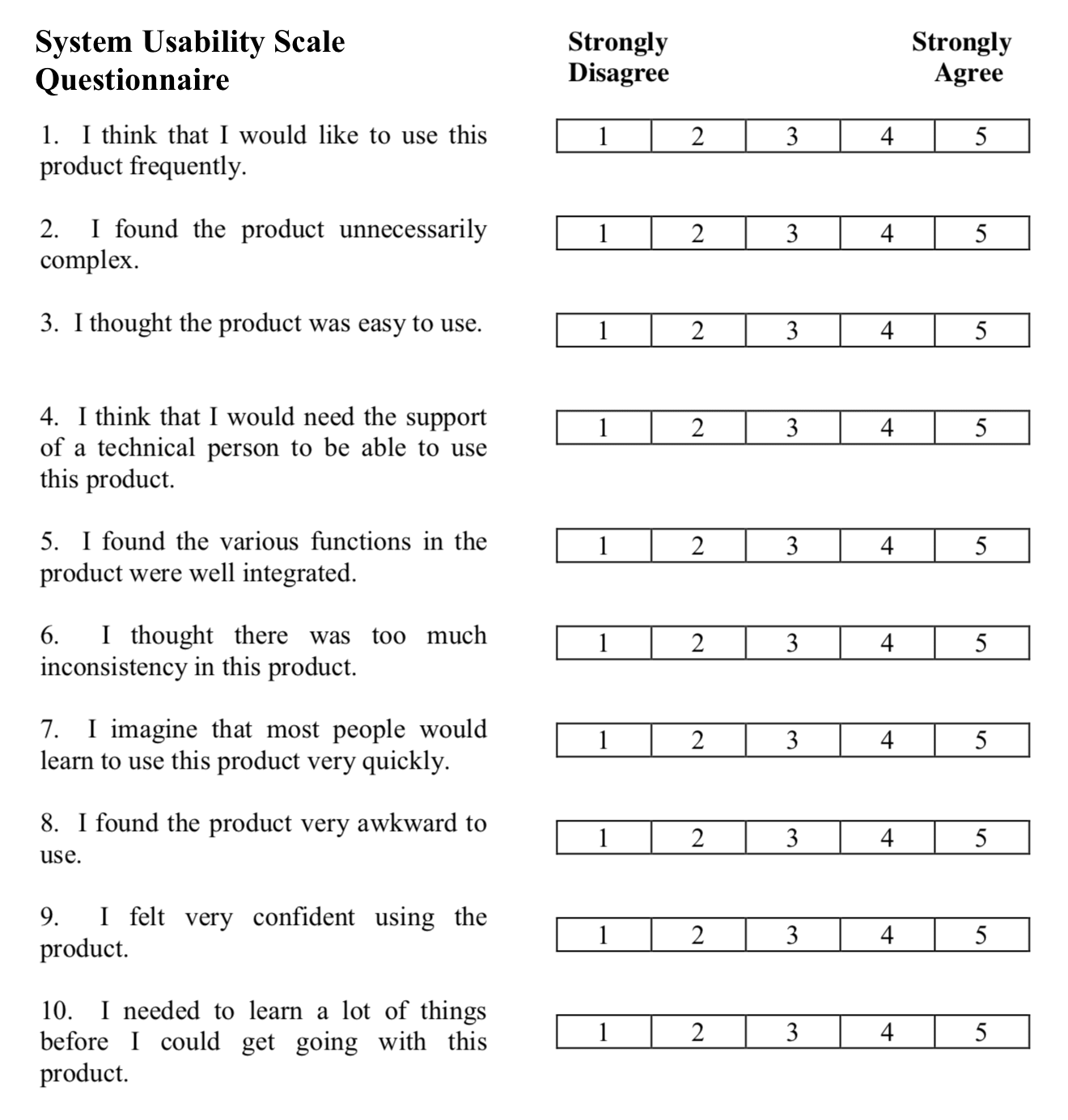


| Acceptability Questionnaire |
| --- |
| 1. What did you like about the FLAGs digital application? |
| 2. What did you dislike about the FLAGs digital application? |
| 3. What are features you wish to be retained in the FLAGs digital application? |
| 4. What do you wish to remove or change in the FLAGs digital application? |
| 5. Are there any other comments you would like to make about the FLAGs digital application? |
